# Development of an acute ovine model of polycystic ovaries to assess the effect of ovarian denervation

**DOI:** 10.3389/fendo.2023.1285269

**Published:** 2023-10-24

**Authors:** W. Colin Duncan, Linda M. Nicol, Rosie O’Hare, Jason Witherington, Jason A. Miranda, Bruce K. Campbell, Jennifer L. Thomas, Michael T. Rae

**Affiliations:** ^1^MRC Centre for Reproductive Health, The University of Edinburgh, Edinburgh, United Kingdom; ^2^Galvani Bioelectronics, Stevenage, United Kingdom; ^3^Division of Child Health, Obstetrics and Gynaecology, The University of Nottingham, Nottingham, United Kingdom; ^4^School of Applied Sciences, Edinburgh Napier University, Edinburgh, United Kingdom

**Keywords:** follicle, tyrosine hydroxylase, polycystic ovary syndrome, sympathetic nerve, gonadotrophin

## Abstract

**Introduction:**

Polycystic ovary syndrome (PCOS) seems to be associated with increased ovarian sympathetic nerve activity and in rodent models of PCOS reducing the sympathetic drive to the ovary, through denervation or neuromodulation, improves ovulation rate. We hypothesised that sympathetic nerves work with gonadotropins to promote development and survival of small antral follicles to develop a polycystic ovary phenotype.

**Methods:**

Using a clinically realistic ovine model we showed a rich sympathetic innervation to the normal ovary and reinnervation after ovarian transplantation. Using needlepoint diathermy to the nerve plexus in the ovarian vascular pedicle we were able to denervate the ovary resulting in reduced intraovarian noradrenaline and tyrosine hydroxylase immunostained sympathetic nerves. We developed an acute polycystic ovary (PCO) model using gonadotrophin releasing hormone (GnRH) agonist followed infusion of follicle stimulating hormone (FSH) with increased pulsatile luteinising hormone (LH). This resulted in increased numbers of smaller antral follicles in the ovary when compared to FSH infusion suggesting a polycystic ovary.

**Results:**

Denervation had no effect of the survival or numbers of follicles in the acute PCO model and did not impact on ovulation, follicular and luteal hormone profiles in a normal cycle.

**Discussion:**

Although the ovary is richly inervated we did not find evidence for a role of sympathetic nerves in ovarian function or small follicle growth and survival.

## Introduction

Polycystic ovary syndrome (PCOS) is a common endocrine disorder affecting 7-8% of women of reproductive age (1). Although there are metabolic aspects of PCOS, ovarian structure and function are key to its diagnosis (2). It is associated with ovarian dysfunction manifested by anovulation, irregular menstrual cycles, increased thecal androgen synthesis and secretion (2), and multiple non-growing, but functional, antral follicles, giving the classic polycystic ovary morphology (3). Ovarian function is regulated by a combination of systemic gonadotrophins and local growth factors (4). Women with PCOS tend to have relatively higher circulating luteinising hormone (LH) concentrations (2) and altered ovarian growth factor profiles (5).

Sympathetic nerves are present within the ovary but the role of ovarian sympathetic innervation remains unclear (6). It has previously been hypothesised that sympathetic nerves act in concert with gonadotrophins to facilitate follicular development and function, including enhancing thecal androgen secretion (7, 8). In rodents hyperstimulation of the ovarian sympathetic nerves increases ovarian noradrenaline concentrations and is associated with a polycystic ovary (PCO) phenotype (9). In a rodent model of PCO induced by juvenile exposure to estradiol valerate (EV) there is evidence for increased ovarian sympathetic activity (10). In the rodent EV PCO model surgical denervation of the superior ovarian nerve decreases ovarian noradrenaline concentrations and induces increased ovulation rate (11, 12). This suggests the sympathetic nervous system may be a therapeutic target in PCOS (13).

There is evidence of an increased sympathetic tone in women with PCOS (14). Women with PCOS have increased muscle sympathetic nerve activity (15) and increased sympathetic drive to the heart (16) and brain (17). Renal sympathetic denervation reduced muscle sympathetic drive as well as improving blood pressure and insulin sensitivity in women with PCOS (18). There is no evidence in women with PCOS whether there is increased sympathetic drive to the ovary and whether ovarian NA concentrations contribute to the development of a PCO morphology.

We hypothesised that the sympathetic nervous system works in parallel with gonadotrophins to facilitate the development of a PCO and that ovarian denervation would improve the polycystic ovarian phenotype. Herein we assessed the effect of ovarian denervation in a large animal model that, unlike rodents, has very similar ovarian function to women. We then developed and validated an acute model of PCO using gonadotrophin manipulation and assessed the effect of denervation on gonadotrophin-driven follicle growth.

## Materials and methods

### Animals

We studied adult Scottish Greyface ewes (*Ovis aries*) weighing 60-75 kg in their third to fifth breeding season. Ewes were housed together in spacious pens with ad libitum hay supplemented with Excel ewe nuts (0.5-1.0kg/day; Carrs Billington, Lancashire, UK) and Crystalayx extra high energy lick (Caltech Solway Mills, Cumbria, UK). All experiments were conducted under Project Licence (PPL60/4401; PCD686E93) from the UK Home Office and underwent institutional ethics review. This work was conducted in accordance with Animals (Scientific Procedures) Act 1986, Galvani Policy on the Care, Welfare and Treatment of Animals Policy 040 and approved by the Galvani Bioelectronics Animal Scientific Review Committee and the GSK Policy on the Care, Welfare and Treatment of Animals. Ovarian sections from an earlier study collected 11 months after whole ovarian cryopreservation and transplantation using Scottish Greyface ewes as described in detail previously (19) were available for analysis.

### Tissue collection

Ewes were killed using a schedule 1 method and ovaries and ovarian vascular pedicles were collected. The vascular pedicles were fixed in Bouin’s solution for 24 hours and transferred to 70% ethanol for subsequent paraffin wax embedding. The ovaries were either: 1) fixed in Bouin’s solution for 24 hours and embedded in paraffin wax for subsequent immunohistochemistry, 2) halved longitudinally and one half fixed in Bouin’s solution and embedded into paraffin wax and the other half snap frozen and stored at -80 °C for subsequent RNA extraction and measurement of intraovarian noradrenaline or 3) fixed in 4% paraformaldehyde for optical projection tomography, depending on the experiment.

### Immunohistochemistry

Mid-ovarian tissue sections cut to 5μm were mounted on permafrost slides. Sections were dewaxed, rehydrated as described previously (20). Antigen retrieval was carried out by pressure cooking for 5 min in 0.01 M citrate buffer, pH 6.0. Sections were washed in water before peroxidase quenching and blocking steps were performed via incubation with 3% H_2_O_2_ for 10 minutes, blocking with avidin and biotin (Vector Laboratories Ltd., Peterborough, UK) and then serum blocking with 20% normal goat serum/5% bovine serum albumin (BSA) in Tris Buffered saline (TBS, 0.05 M Tris pH 7.4, 0.85% NaCl). Slides were washed in TBS between treatments, then in TBS containing 0.025% Triton X-100 (TBS-T) prior to serum block and antibody incubation.

The primary antibody diluted in serum block (mouse anti-tyrosine hydroxylase 1:1000 (Sigma-Aldrich Ltd, Dorset, UK), mouse monoclonal anti-Ki67 1:100 (Novocastra, Newcastle, UK) (1:1000) or rabbit polyclonal anti-caspase 3 1:100 (Cell signalling, MA, USA)) was applied to sections and incubated overnight at 4°C. Slides were washed in TBS-T and the secondary antibody (biotinylated goat anti-mouse or goat anti-rabbit (Vector Laboratories, Peterborough, UK) diluted 1:500 in serum block) was applied to slides for 1 hour. Slides were washed in TBS-T followed by Vectastain ABC Elite tertiary complex (PK-1600 series; Vector Laboratories) for 1 hour after which 3,3′-diaminobenzidine (Dako, Cambridge, UK) was applied for 3 minutes to visualise binding. Sections were then counterstained with haematoxylin and mounted using Pertex mounting medium (Cellpath, Newtown, Powys, UK). Negative controls were non-specific mouse or rabbit serum of equivalent immunoglobulin concentrations in place of the primary antibody.

### Immunofluorescence

Dual labelled tissue sections were prepared for confocal microscopy following the immunohistochemistry protocol described above, with the following adjustments. The peroxidase wash step was omitted and the slides were permeabilised normally through a series of two five-minute washes in TBS-T. After an incubation time of one hour in 10% normal goat serum, the endothelial antibody (Rabbit monoclonal anti-CD31, Vector Laboratories) and mouse anti-tyrosine hydroxylase antibody were diluted together at a concentration of 1:100 and 1:200 respectively in TBS, before being added to the slides and incubated overnight at 4°C.

After washing in TBS with 0.01% Tween 20 (Sigma-Aldrich, UK) the slides were incubated with biotinylated goat-anti-rabbit secondary antibody diluted in 10% NGS at a concentration of 1:500 for one hour at room temperature. After washing in TBS-Tween 20 (0.01%) the slides were incubated with Dylight^®^ 594 (Thermo-Fisher, UK) and goat-anti-mouse IgG secondary antibody conjugated to Alexaflour^®^ 488 (Invitrogen, UK), each at 1:100 in 10% NGS. Slides were incubated in the dark at room temperature for two hours in a humidity chamber. After washing with TBS-Tween 20 in the dark slides were mounted in an aqueous solution containing 4’6-diamidino-2-phenyllindole (DAPI), and stored for 12 hours at 4°C, prior to visulisation on a Zeiss LSM 880 AxioObserver Z1 confocal fluorescent microscope (wavelengths: 405 nm, 488 nm, 594 nm, laser power set at 2%).

### Analysis of tissue sections

Two examiners, blinded to treatment, graded the immunohistological staining (based on area of staining) of tyrosine hydroxylase independently and the scores were averaged for whole ovary sections. Each ovary section was examined and graded out of four, with zero indicating no staining present and four indicating abundant staining throughout the tissue. Spatiotemporal examinations of vessel and nerve relationships were made using a Zeiss LSM880 Confocal Microscope. Images were captured to illustrate this at 20x magnification, and 63x magnification with oil.

Two independent examiners, blinded to treatment, also independently counted the number of follicles from a standardised mid-section of the ovary (21) as well as the number of preantral follicles. Follicles were classified as preantral if they did not show any antral cavity and antral if they showed a clear fluid-filled antrum (>500 µm). Immunohistochemical staining of whole ovary sections stained for proliferation (Ki67) and atresia (activated caspase 3) were blindly examined by two independent expert examiners. Each antral follicle was examined and staining was divided into two classifications, positive (clearly positive immunostaining present in multiple cells) and negative (scant/absent immunopositive cells). Number of follicles per classification was used for proportional analysis as described previously (21).

### Ovarian nerve ablation

A mini-laparotomy was performed with sterile technique under general anaesthesia, induced using isoflourane (Isoflo, Abbott Animal Health, Maidenhead, UK). A small paramedian incision exposed the ovaries. To avoid non-specific ovarian damage we specifically targeted the nerves in the ovarian neurovascular pedicle. Needlepoint diathermy using monopolar coagulation current (Surgitran, STW-100) (22) was used to coagulate around the ovarian vessels, in the regions where sympathetic nerves had been identified, leaving blood vessel integrity intact.

### Intraovarian noradrenaline measurements

A 3 mm^3^ sample from the ovarian cortex at the lateral edge of the ovary was used to measure intraovarian noradrenaline (NA) concentrations. It was weighed and homogenised in lysis buffer (0.01N HCl, 1 mM EDTA, 4mM Na_2_S_2_O_5_). Lysate was spun at 5000 rpm at 4 °C for 10 min and the supernatant analysed. Quantification was carried out using the competitive NA ELISA kit (IMMUSMOL, Pessac, France) as described previously (23) following the manufacturer’s instructions. NA was extracted using a cis-diol-specific affinity gel, acylated and then derivatised enzymatically. The antibody bound to the solid phase was detected using an anti-rabbit IgG-peroxidase conjugate and tetramethylbenzidine (TMB) as a substrate. The reaction is monitored at 450 nm. The sensitivity was 2 pg/ml, and the intra and interassay CVs were <9%. The cross reactivity found was 0.14% for adrenaline and 1.8% for dopamine.

### Plasma hormone measurements

Plasma estradiol and progesterone concentrations were measured using a commercial ELISA following the manufacturer’s instructions on a Cobas E411 immunoanalyser (Roche, Mannheim, Germany). The progesterone assay (Cobas progesterone II) has a sensitivity of 0.48 nmol/l. The cross reactivity with related steroids is <1%. The estradiol assay (Cobas Estradiol III) has a sensitivity of 11 pmol/l. Apart from 6α-OH estradiol the cross reactivity of related steroids is <1%. Both assays have CVs <10%.

### Quantitative real time PCR

RNA was extracted from tissue using RNeasy mini spin columns following manufacturer’s protocol and concentration measured using NanoDrop 1000 Spectrophotometer as described previously (20). Complimentary DNA (cDNA) was synthesised from 200 ng RNA in accordance with manufacturer’s protocol (Applied Biosystems, California, USA). Subsequently, qRT-PCR was performed using SYBR Green. Real-time PCR reactions were carried out in duplicate 10 µl reactions, negative controls consisted of cDNA reaction without reverse transcriptase and a reaction replacing cDNA with nuclease-free water. Melt curve analysis revealed a single amplicon in all cases. GAPDH has been reported as a suitable internal control for ovarian stromal gene expression (24) and target gene expression was analysed relative to GAPDH and quantified using the DCt method.

Primer3 Input version 0.4, online software, was used to design forward and reverse primers from DNA sequences obtained from Ensembl Genome Browser. Sequences were checked for specificity using Basic Local Alignment Search Tool and validity confirmed as previously described (25). The primers 5’-3’ were: CCN2: TGCCCTCGCAGCTTACC and CTTGGAACAGGCACTCCACT; VEGF: TCTTCAAGCCATCCTGTGTG and TGCATTCACATTTGTTGTGC; NGF: CTGGCCACACTAAGGTGCATA and GCTGCCTGTATGCCGATCAA; IGF1: CATCCTCCTCGCATCTCTTC and CTCCAGCCTCCTCAGATCAC; FGF2: ACTTTAAGGACCCCAAGCGG and AGTTTGATGTGAGGGTCGCT; GAPDH: GGCGTGAACCACGAGAAGTATAA and AAGCAGGGATGATGTTCTGG.

### Development of acute model of PCOS

**Figure 1:**
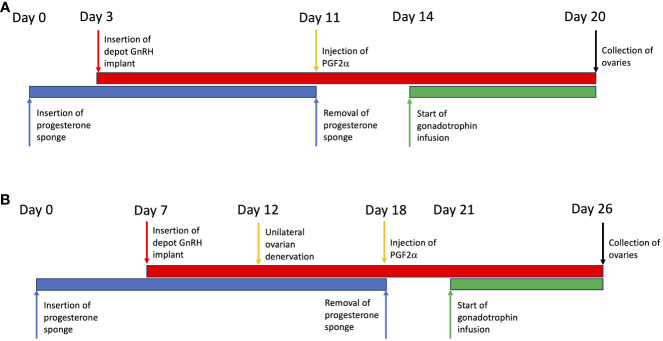
Illustration of the protocols used in the development and manipulation of a polycystic ovary. **(A)** Validation of the model. **(B)** Using the model to test the effect of ovarian denervation.

Intravaginal progestogen-impregnated sponges (60 mg medroxyprogesterone acetate per sponge; Intervet Laboratories Ltd, Cambridge, UK) were inserted into ewes (n=6) and then 3 days later gonadotrophin releasing hormone implants (GnRH; Suprelorin; 4.7 mg Deslorelin acetate; Virbac, UK) were inserted, through large bore needles, for pituitary suppression. Eleven days later the sponge was removed and the sheep were given an injection of prostaglandin F2α (PG; 100 mg Cloprostenol; Estrumate; Coopers Animal Health Ltd, Crewe, Cheshire, UK) to ensure luteolysis of any residual corpora lutea and prepare for an artificial follicular phase. Three days later the jugular vein of the sheep was canulated and infusions started. FSH (Folltropin; Vetoquinol UK Ltd, Buckinghamshire, UK) given at 1mg/hour via jugular catheter using Graseby MS 16A syringe drivers; Luteinizing Hormone (LH; ovine LH NIADDK-oLH-27; Dr. A.F. Parlow, Harbor-UCLA-Medical Center, Torrance, CA) given as 4 hourly pulses via the jugular catheter (18 µg/pulse) using Zyklomat pulse infusion pumps, for 6 days. Sheep were given either physiological FSH concentrations only with baseline endogenous LH (n=3) or physiological FSH + additional exogenous LH infusions (n=3) (**Figure 1A**). At end of infusions the left ovary from each animal was processed for optical projection tomography (OPT) scanning as described below.

### Testing the effect of denervation of acute model of PCOS

Progesterone sponges were inserted in ewes (n=6) and 7 days later GnRH implants were inserted as described above. Five days later mini-laparotomy was performed as described above followed by unilateral diathermy needle denervation to the left ovary was performed, allowing the right ovary to serve as an internal control. Six days later sponges were removed and PG injections were given as described above. Sheep were cannulated 3 days later and infusions started as described above with FSH (1 mg/hour) (n=3) or FSH+LH (FSH 1 mg/hour; LH 4 hourly pulses of 18 µg/pulse) (n=3) for 6 days (**Figure 1B**). At the end of infusions both ovaries were processed for OPT scanning as described above.

### Optical projection tomography

Ovaries processed for OPT were fixed in 4% paraformaldehyde overnight, washed 4 x 30 minutes Phosphate Buffered Saline (PBS), then 30 minutes each in 30%, 70%, 90%, 100% Ethanol. They were transferred to Methanol for 2 hours, then into fresh Methanol and stored at 4°C until processed for scanning. Ovaries were attached to mounting blocks, and then immersed in BABB (2 parts Benzyl Benzoate, 1 part Benzyl Alcohol) until cleared sufficiently for scanning. Cleared ovaries were scanned in a calibrated Bioptonics 3001 tomograph (Bioptonics, UK). Dataviewer (Version 1.5.2.4 Release, July 2015) was used to combine the scans into 2D and 3D models to then quantify the size and number of each follicle throughout each ovary. After scanning ovaries were returned to methanol to remove BABB, then washed 30 min each in 100%, 90% and 70% Ethanol. They were stored in 70% Ethanol prior to embedding in paraffin wax for sectioning.

### Statistical analysis

Statistical analysis was conducted using GraphPad Prism 8.0 (GraphPad Software, San Diego, CA) with P<0.05 considered statistically significant. Proportional contingency table analysis was measured by Fisher’s exact test for 2x2 tables and Chi squared for larger tables. After checking for normality and similar variances two column comparisons were examined using unpaired two-tailed t-tests if the data was parametric and Mann Whitney U tests if not parametric or normally distributed. Correlation was assessed using Spearman co-efficient of correlation.

## Results

### Sympathetic innervation of the ovine ovary

**Figure 2:**
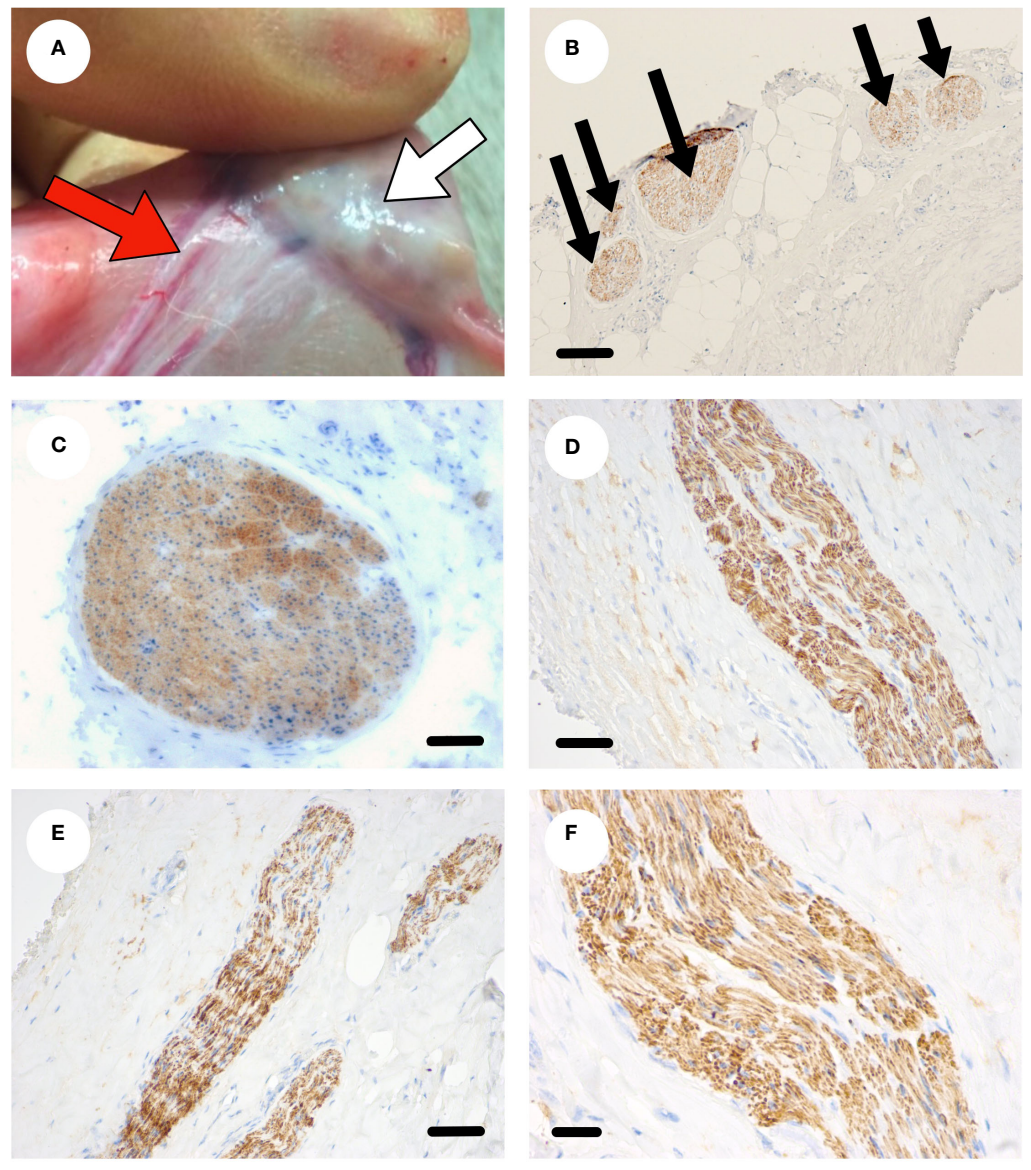
Sympathetic nerve supply to the ovine ovary. **(A)** Photograph of an ovine ovary (white arrow) *in situ* highlighting the neurovascular pedicle (red arrow). **(B)** Transverse section through the ovarian pedicle stained with tyrosine hydroxylase (brown) showing several discrete sympathetic nerves within the pedicle. **(C)** A large sympathetic nerve (brown) in the neurovascular pedicle. **(D-F)** Smaller sympathetic nerves (brown) within the pedicle. Scale bar **(B–D)** = 50 µm, **(E, F)** = 20 µm.

The ovaries and ovarian pedicle of Scottish Greyface sheep (n=6) were examined macroscopically (**Figure 2A**). The vascular bundle, consisting of the ovarian artery closely intertwined with the utero-ovarian venous plexus enters the hilum of the ovary through the ovarian pedicle (**Figure 2A**). Microscopic examination of the vascular pedicle showed sympathetic nerves running with the ovarian vessels (**Figure 2B**). Sometimes there was one discrete nerve in the ovarian pedicle (**Figure 2C**) but commonly there were several smaller nerves (**Figures 2D-F**) from 50 µm to 350 µm in diameter. There were no other neurovascular entry points to the ovary outside the hilum and ovarian pedicle. In each case at least one nerve ≥50 µm could be identified consistently with the ovarian artery. Sympathetic innervation of the ovine ovary runs along the vascular bundle in the ovarian pedicle.

### The location of sympathetic nerve fibres within the ovary

**Figure 3:**
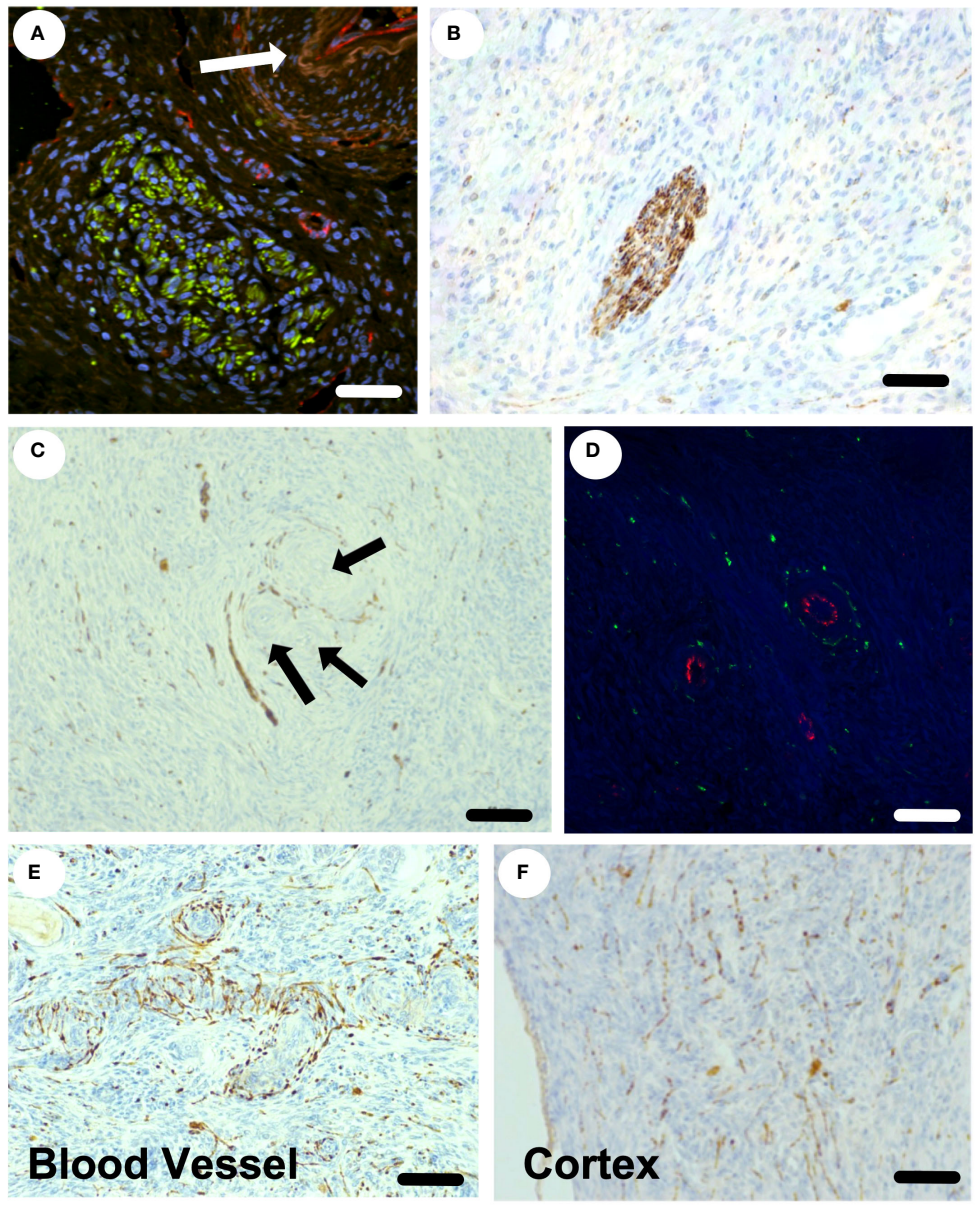
Sympathetic nerve supply within the ovine ovary. **(A)** Confocal staining of the hilar region of the ovary stained for tyrosine hydroxylase (sympathetic nerves) in green and CD-31 (endothelial cells) in red. An arteriole is highlighted by the white arrow. **(B)** A discrete sympathetic nerve (brown) within the ovarian stroma. **(C)** Nerves (brown) seen around blood vessels and a small preantral follicle (arrows). **(D)** Small arterioles with endothelial staining (red) with clear sympathetic nerves (green) surrounding in transverse view. **(E)** The plexus of sympathetic nerves (brown) around an arteriole in longitudinal view. **(F)** The presence of sympathetic nerves throughout the ovarian cortex and around small preantral follicles. Scale bar = 50 µm.

The sympathetic nerves enter the ovary at the hilum as discrete nerves next to blood vessels (**Figure 3A**) and can be seen as discrete nerve fibre bundles within the ovarian medulla (**Figure 3B**). Sympathetic nerves branch further within the ovary and are associated with arterioles and small blood vessels (**Figures 3C-E**). Nerve fibres are also seen in the cortical regions of the ovary, in the vicinity of primordial and primary follicles, that are not associated with blood vessels (**Figure 3F**). Overall, 40% of nerves identified within the ovary were not associated with blood vessels. There is sympathetic innervation that is independent from blood vessels around primordial, primary and secondary follicles in the ovarian cortex.

### Regeneration of ovarian nerves after denervation

**Figure 4:**
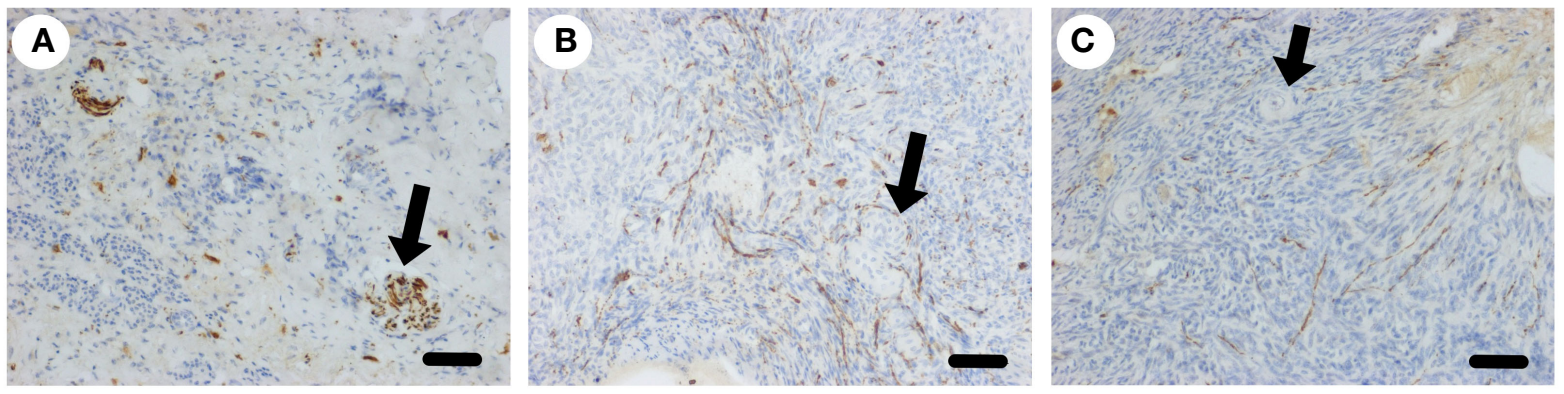
Sympathetic nerve supply to the ovary after transplantation. **(A)** Larger nerves (arrow) stained for tyrosine hydroxylase (brown) in the hilar region of the ovary post transplantation. **(B)** Plexus of sympathetic nerves (brown) around blood vessels in the medulla (arrow). **(C)** Sympathetic nerves in the cortex close to primordial follicles (arrow). Scale bar = 50 µm.

If sympathetic nerves have a physiological role in ovarian function it would be expected that they would regenerate after ovarian denervation. We examined sympathetic nerves in the ovine ovary after oophorectomy, whole ovary cryopreservation and ovarian transplantation (18). The ovaries were disconnected from the neurovascular bundle and thus denervated. After transplantation back onto the ovarian pedicle the ovaries became functional (18). Ten months after transplant histological analysis of ovaries (n=4) showed that all ovaries had discrete nerves (50 µm) at the hilum (**Figure 4A**) and a normal distribution of nerves throughout the ovarian stroma, including association with arterioles (**Figure 4B**) and cortical tissue independent of blood vessels (**Figure 4C**). After denervation the ovine ovary is reinnervated in situ.

### Acute denervation of the ovine ovary

**Figure 5:**
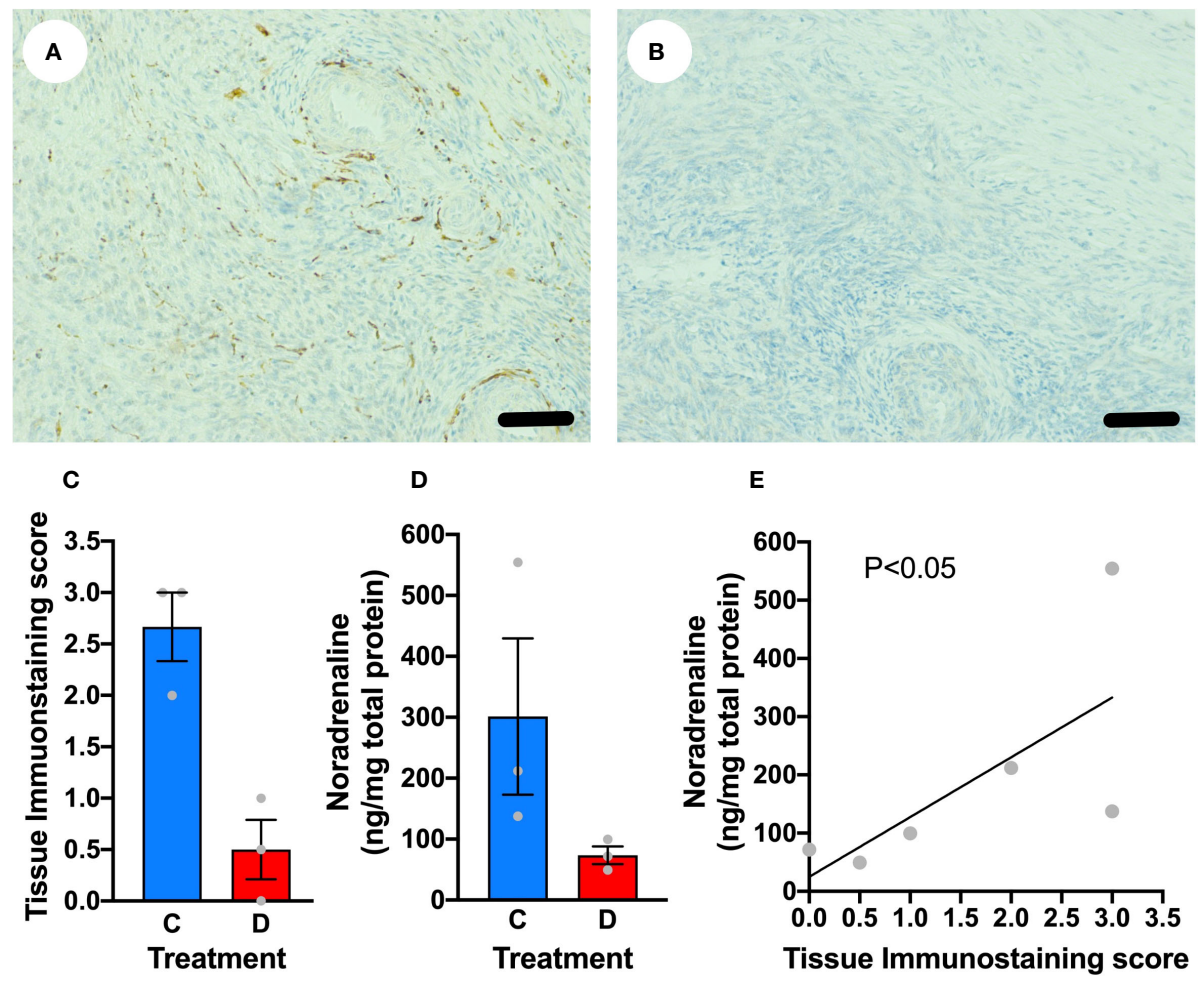
Denervation of the ovary using needlepoint diathermy. **(A)** Ovarian stroma stained for tyrosine hydroxylase (brown) highlighting sympathetic nerves that was histoscored blindly as 3. **(B)** Contralateral ovary stained for tyrosine hydroxylase after diathermy for denervation showing no specific immunostaining, with a histoscore of 0. **(C)** Blinded tissue score for immunostaining for tyrosine hydroxylase in control **(C)** ovary and diathermy **(D)** ovary. **(D)** Tissue noradrenaline concentrations in control (C) ovary and diathermy (D) ovary. **(E)** Significant correlation between noradrenaline concentrations and tissue immunostaining score for noradrenaline. Scale bar = 50 µm.

In order to determine if we could acutely denervate the ovine ovary, ewes (n=3) underwent laparotomy and unilateral denervation in the mid-follicular phase using monopolar needle micro-diathermy of the putative ovarian nerves within the neurovascular bundle leaving the vasculature intact. After 21 days the sheep were killed and the ovaries examined. There was a loss of sympathetic nerve fibre immunostaining within the treated ovary compared to the contralateral control ovary (**Figures 5A-C**). In addition, there was reduction in intraovarian noradrenaline concentrations (**Figure 5D**) that correlated with tissue immunostaining score (r=0.8407; P<0.05; **Figure 5E**). Needle diathermy of the sympathetic nerves in the ovarian pedicle can be used to acutely denervate the ovary.

### The acute effects of ovarian denervation

**Figure 6:**
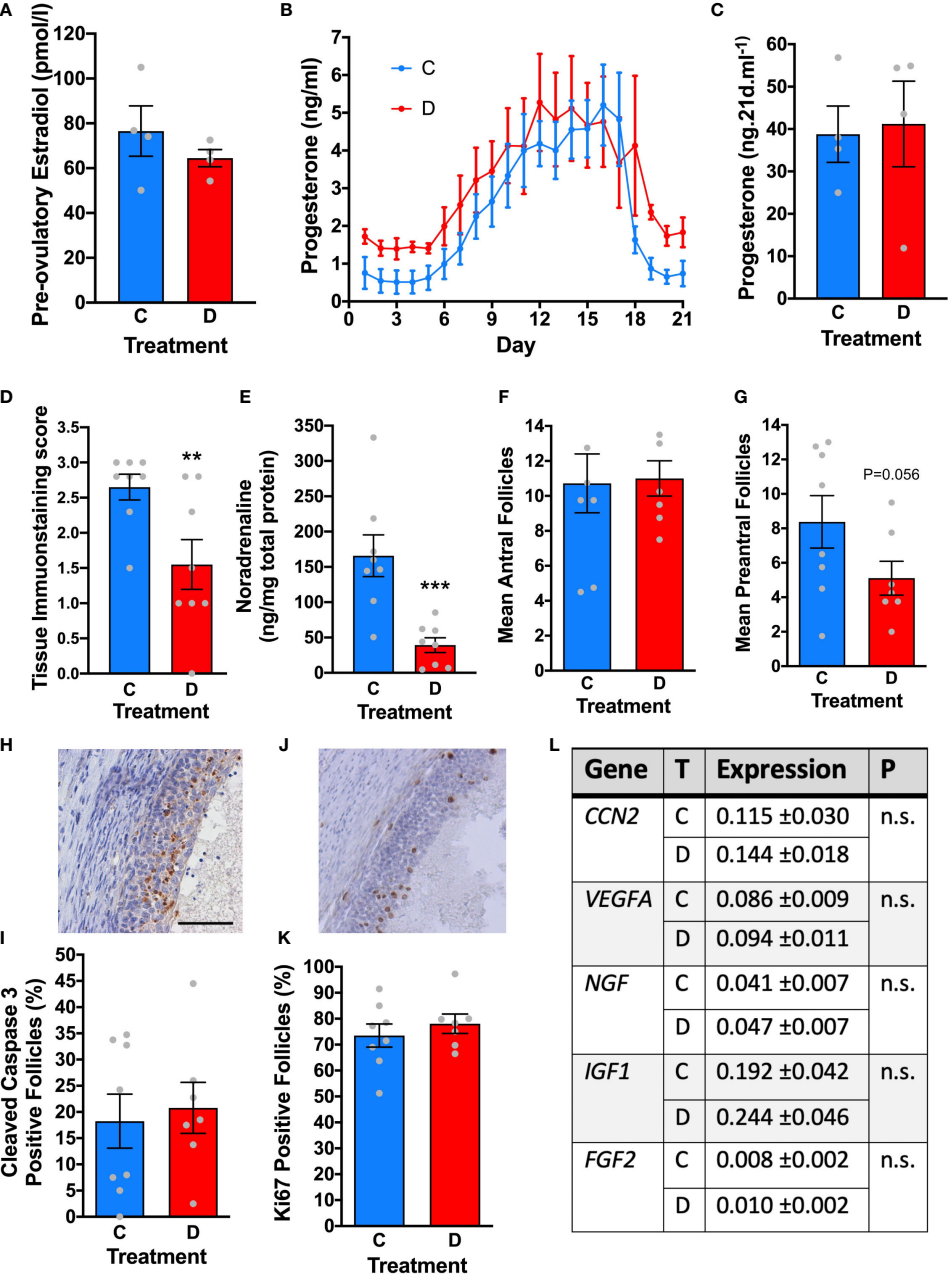
The effect of denervation on ovarian structure and function. **(A)** Peak estradiol before ovulation in control (C, n=4) and after ovarian denervation (D; n=4). **(B)** progesterone dynamics across the luteal phase after ovulation in control (C) and ovarian denervation (D) sheep. **(C)** Total progesterone secretion across the luteal phase in control (C, n=4) and after ovarian denervation (D; n=4). **(D)** Significant reduction in tissue immunostaining score and **(E)** ovarian noradrenaline concentrations after denervation (each ovary is analysed separately). **(F)** No significant difference in number of antral follicles or **(G)** preantral follicles in representative mid ovarian tissue section. **(H)** Representative immunostaining for cleaved caspase-3 (brown) identifing follicular atresia. **(I)** Quantification of antral follicles positive for cleaved caspase-3 in control ovaries (C) and after ovarian denervation (D). **(J)** Representative immunostaining for Ki67 (brown) identifying growing follicles. **(K)** Quantification of antral follicles positive for Ki67 in control ovaries (C) and after ovarian denervation (D). (**L)** Transcript abundance in ovarian stroma for key ovarian growth factors in control ovaries (C) and after ovarian denervation (D). ** P<0.01, *** P<0.005, n.s, not significant, scale bar = 50 µm.

A separate cohort of ewes were randomised to either bilateral denervation using micro-diathermy (n=4) or a sham procedure without diathermy (n=4). The hormonal profiles of the ewes were then examined daily over an ovarian cycle and ovaries were collected 21 days later and at that stage we examined intraovarian noradrenaline concentrations to confirm ongoing denervation during the experiment. In the follicular phase there was no difference in estradiol concentrations (**Figure 6A**). There was no effect of denervation in the timing of ovulation, post-ovulatory progesterone concentrations and luteolysis (**Figures 6B, C**). Analysis of the tissue immunostaining score for sympathetic nerves (P<0.01; **Figure 6D**) and intraovarian noradrenaline concentrations (P<0.005; **Figure 6E**) confirmed ovarian denervation after micro-diathermy.

Follicles were counted in a representative mid-ovarian section from each ovary. There were no differences in antral follicle numbers (**Figure 6F**) although there was a strong trend to less preantral follicles after diathermy but this didn’t reach statistical significance (P=0.056; **Figure 6G**). There were no differences in proportion of atretic antral follicles, assessed by cleaved caspase 3 expression (**Figures 6H, I**) or growing antral follicles, assessed by Ki67 localisation (**Figures 6J, K**). In addition, there were no differences in the ovarian transcript abundance of *CCN2*, *VEGFA*, *NGF*, *IGF1* and *FGF2* (**Figure 6L**). Acute denervation does not have any impact on follicular growth, ovulation and luteolysis or on the survival of antral follicles.

### Development of an acute ovine model of PCOS

**Figure 7:**
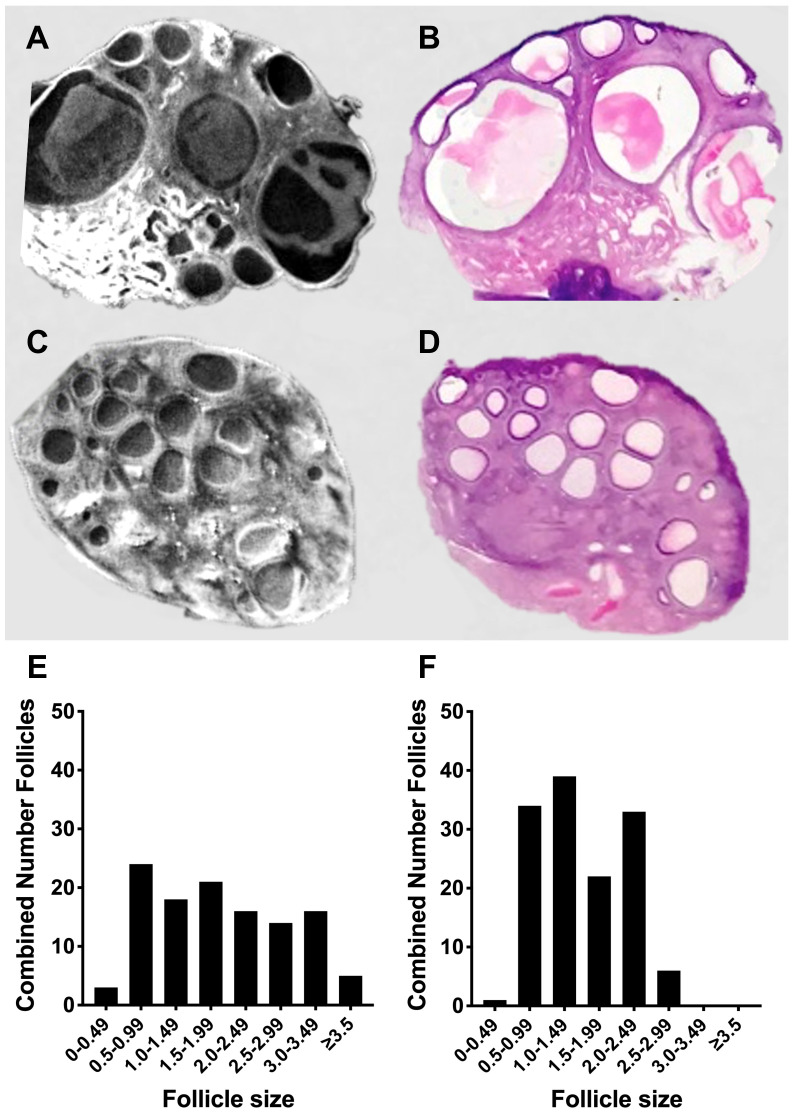
Acute modelling of PCO. **(A)** Static image of optimal tomography whole ovarian scan with **(B)** the same area of the ovary after tissue sectioning and haematoxylin and eosin staining after FSH infusion with low LH. **(C)** Static image of optimal tomography whole ovarian scan with **(D)** the same area of the ovary after tissue sectioning and haematoxylin and eosin staining after FSH infusion with high pulsatile LH. **(E)** Cumulative follicles in the whole ovary based of size after FSH infusion with low LH (n=3). **(F)** Cumulative follicles in the whole ovary based of size after FSH infusion with high pulsatile LH (n=3).

To develop an acute ovine model of PCOS, in normal cycling sheep, we first synchronised the sheep with progesterone sponges then switched off the hypothalamic pituitary ovarian axis using a GnRH agonist (n=3). Sheep were given an infusion of FSH with baseline endogenous LH (Control) or with additional high dose pulsatile LH (PCOS-like). After seven days the ovaries were collected and analysed by optical tomography, which allows the whole ovary to be viewed in real-time digital sections to accurately count and measure all the antral follicles (**Figures 7A, C**). These follicles could be identified in subsequent tissue sections, and had a normal follicular structure including healthy granulosa cell and theca cell layers (**Figures 7A, B**), after further fixation and sectioning after OPT was complete. There was a different pattern of follicles in the PCO-like ovaries (P<0.0001) with an increased number of smaller and a reduced number of larger follicles, suggesting a polycystic morphology (**Figures 7C, D, F**). Gonadotrophin manipulation can facilitate the acute development of polycystic ovaries.

### The effect of denervation in the acute PCOS model

**Figure 8:**
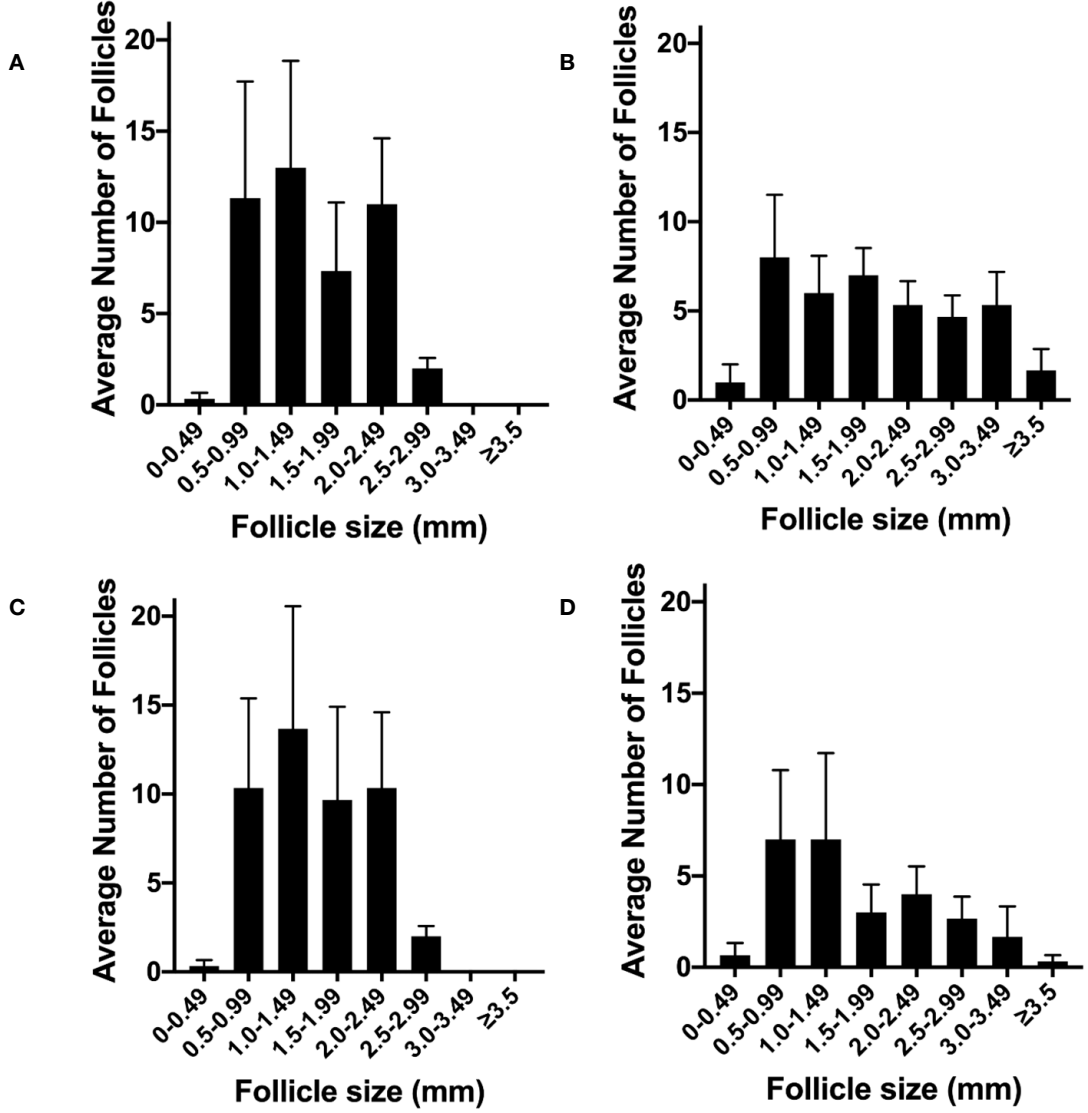
The effect of ovarian denervation on acute model of PCO. **(A)** Average number of antral follicles per ovary after FSH infusion with high pulsatile LH (n=3), **(B)** or after FSH infusion with low LH (n=3), after sham procedure. **(C)** Average number of antral follicles per ovary after FSH infusion with high pulsatile LH (n=3), **(D)** or after FSH infusion with low LH (n=3), after bilateral ovarian denervation procedure.

After bilateral denervation we then assessed the effects of gonadotrophin infusion to create the acute PCOS-like model. There was a difference in the pattern of follicles in the control high dose pulsatile LH PCOS-like sheep (**Figure 8A**) compared to the control low LH sheep (**Figure 8B**). However, denervation showed no difference in the pattern or number of follicles in the high LH PCOS-like sheep (**Figure 8C**) or the control low LH sheep (**Figure 8D**). Denervation had no effect on gonadotrophin action in the development of a PCO ovary.

## Discussion

We have shown that there is dense sympathetic innervation in the ovine ovary that is not only associated with blood vessels but also seen around the avascular small follicles. Sympathetic denervation, confirmed by intra-ovarian sympathetic nerve immunostaining and NA measurement, has no effect on antral follicle growth, ovulation or luteolysis. In addition, it had no effect on gonadotrophin action in follicular development. We recreated a polycystic ovarian morphology using gonadotrophin manipulation to test the effect of sympathetic denervation. There was no effect on the acute antral follicle response. This has narrowed down the potential roles of the sympathetic nervous system in the ovary.

Sympathetic innervation of the ovary is seen in multiple species including rodents (26), ruminants (27), non-human primates (28) and women (29). In polyovulatory species such as rodents and pigs there is very clear innervation with easy identification of the superior ovarian nerve (23, 30). We hypothesised that this may suggest that sympathetic innervation may protect follicles from atresia, or promote early follicular development, and this may have a role in the development of a polyfollicular (polycystic) ovary. We used a clinically realistic ovine model as the sheep has a robust track record in clinically relevant ovarian research (19, 21, 31).

We surmised that total severance of the sympathetic nerves entering the ovary would occur during oophorectomy (19). If ovarian innervation was important for normal ovarian function then nerves would regrow into the ovary after auto-transplantation. The presence of a normal intraovarian sympathetic nerve distribution after resumption of ovarian activity post transplantation does suggest a relevant role for ovarian sympathetic innervation. It has been postulated that ovarian innervation is involved in regulating local gonadotrophin action, either directly (7, 8) or though regulation of vascular blood flow. Indeed, there is some evidence that cells within ovarian follicles have some features of nerve cells (32) and cells within the follicle express receptors to NA (33). In rodents, stimulation of the sympathetic nerves increased the number of antral follicles (9). This supports a role for the sympathetic nervous system in supporting follicular growth. Unfortunately, our assay for testosterone was not sensitive enough to allow us to examine the effect of denervation on androgen secretion. It has been suggested that sympathetic nerves facilitate LH-dependent androgen secretion (8), and it is the androgens that are important in the development of a polycystic ovary (34). We cannot say if denervation reduced androgens but showed there was no effect on estrogen levels and importantly no effect on the development of a polycystic ovary induced by increased LH concentrations.

We were able to denervate the ovary and examine what happened during the follicular and luteal phase of a cycle. Follicular growth and ovulation occurred normally. Importantly luteolysis also occurred normally. As luteolysis involves a vascular counter-current between the uterus and the ovary (35) this would suggest there was no acute effect on the vasculature that might have delayed luteolysis (19). This suggests that in the absence of sympathetic stimulation gonadotrophins can independently drive mid-late follicular growth and ovulation.

If the sympathetic nervous system can augment gonadotrophin action in the ovary, and promote follicular survival, this might facilitate the development of a polycystic ovary in PCOS. Women with PCOS have increased LH action within the ovary (2). Manipulation of LH concentrations can impact on follicular growth and development (36). We hypothesised that increasing LH action in the ovary would increase the number of follicles but block the growth of large antral follicles, developing a polycystic ovary. Using OPT allows every antral follicle within the whole ovary to be measured and counted. Driving the ovary with increased LH concentrations resulted in a different pattern of follicular growth and the development of a macroscopic polycystic ovary.

This acute model of PCO allowed us to determine the effects of sympathetic denervation on LH action in the development of a polycystic ovary. We hypothesised that after denervation the polycystic ovary would have less and larger follicles. Elegant rodent studies involving reducing sympathetic innervation improved ovarian function in an induced polycystic ovary phenotype (29). There was no acute effect on ovarian morphology and the denervated ovary developed the same PCO morphology is response to gonadotrophin manipulation as the innervated ovary. Overall this suggests that sympathetic nerves in a large animal, human-like, ovary are not involved in the gonadotrophin dependent phase of follicular growth (4). It remains possible that these nerves do have a function as growth and/or survival factors for smaller gonadotrophin independent follicles. Denervation showed a trend towards a reduction in preantral follicles in the short term.

In summary we have developed an acute model of PCO ovarian morphology in an ovine model by manipulating gonadotrophins, which may have future utility in terms of separation of metabolic aspects from ovarian aspects of this syndrome. One benefit of this model was that it allowed us to investigate the role of the sympathetic nervous system in the regulating gonadotrophin action in the follicle. Gonadotrophins are the master regulator of the gonadotrophin dependent follicle and there does not seem to be any significant neural contribution. However, it remains likely that sympathetic action is involved in ovarian function in concert with gonadotrophins. The involvement of sympathetic nerves in the polycystic ovary, and a physiological effect of testosterone secretion, remains possible but that gonadotrophin action is the fundamental driver of ovarian structure and function. There may be effects before gonadotrophins take over follicular growth and development and possible effects on local androgen production that are not able to be ascertained in this model. This suggests that longer term experiments, perhaps using a clinically realistic prenatally programmed ovine model of PCOS (20, 24) would need to be used to dissect the role of the sympathetic nervous system on the survival of smaller follicles, follicular steroidogenesis and the development of the polycystic ovary.

## Data availability statement

The raw data supporting the conclusions of this article will be made available by the authors, without undue reservation.

## Ethics statement

The animal study was approved by The UK Home Office, The University of Edinburgh Institutional review and Galvani Bioelectronics Animal Scientific Review Committee. The study was conducted in accordance with the local legislation and institutional requirements.

## Author contributions

WCD: Conceptualization, Formal Analysis, Funding acquisition, Methodology, Project administration, Writing – original draft, Writing – review & editing. LN: Data curation, Formal Analysis, Writing – review & editing. RO’H: Formal Analysis, Investigation, Writing – review & editing. JW: Funding acquisition, Resources, Writing – review & editing. JM: Funding acquisition, Resources, Writing – review & editing. BC: Resources, Writing – review & editing. JT: Formal Analysis, Writing – review & editing. MR: Funding acquisition, Methodology, Supervision, Writing – review & editing.
